# Hyperbaric oxygen therapy promotes neurogenesis: where do we stand?

**DOI:** 10.1186/2045-9912-1-14

**Published:** 2011-06-27

**Authors:** Jun Mu, Paul R Krafft, John H Zhang

**Affiliations:** 1 Department of Physiology, Loma Linda University School of Medicine, 11021 Campus Street, Loma Linda, CA 92354, USA; 2 Department of Neurology, The First Affiliated Hospital of Chongqing Medical University, 1 Yi Xue Yuan Road, Chongqing 400016, China; 3 Department of Neurology, Loma Linda University School of Medicine, 11234 Anderson Street, Loma Linda, CA 92354, USA

## Abstract

Neurogenesis in adults, initiated by injury to the central nervous system (CNS) presents an autologous repair mechanism. It has been suggested that hyperbaric oxygen therapy (HBOT) enhances neurogenesis which accordingly may improve functional outcome after CNS injury. In this present article we aim to review experimental as well as clinical studies on the subject of HBOT and neurogenesis. We demonstrate hypothetical mechanism of HBOT on cellular transcription factors including hypoxia-inducible factors (HIFs) and cAMP response element binding (CREB). We furthermore reveal the discrepancy between experimental findings and clinical trials in regards of HBOT. Further translational preclinical studies followed by improved clinical trials are needed to elucidate potential benefits of HBOT.

## Introduction

Neurogenesis is defined as generation of neurons within the brain. In adults, neurogenesis occurs primarily in two brain regions: the subventricular (SVZ) and the subgranular zone (SGZ) of the hippocampal dentate gyrus (DG). Injury to the central nervous system (CNS) including trauma, cerebral ischemia and epileptic seizures have been reported to induce neurogenesis, and surviving cells may be functionally integrated into existing neural circuits [1]. Consequently, further endogenous promotion of neurogenesis may hold promise for restoration of cerebral functions after CNS injury.

Hyperbaric oxygen therapy (HBOT) refers to the medical use of oxygen at a level higher than atmospheric pressure. Initially, indicated for decompression illness it has been further applied to clinical conditions including crush injury, diabetic foot, skin grafts, thermal burns and to several neurological diseases [2]. Elevation of partial oxygen pressure in the body, leads to increased oxygen transport capacity of erythrocytes, facilitating peripheral regeneration processes (e.g. angiogenesis).

It has been suggested that HBOT exerts neuroprotective effects through a variety of mechanisms, including the activation of cellular transcription factors [3]. However, due to inconsistent results and few clinical trials, HBOT for neurologic disorders has not yet been approved by the FDA. Further preclinical studies are needed to clarify the effect of HBOT on neurogenesis and to ensure a successful translation to clinical trials.

## Literature Review

Publications were identified by PubMed/Medline and Web of Science, using the following keywords: neurogenesis, hyperbaric oxygen, ischemia, proliferation and BrdU. All publications, languages and subsets were explored. Results from previous studies were summarized into the following four categories: hypoxic-ischemic encephalopathy (HIE) (Table 1), vascular dementia (Table 2), permanent middle cerebral artery occlusion (MCAo) (Table 3) and human mesencephalic neural progenitor cells (hmNPCs) (Table 4).

**Table 1:** Hypoxic-ischemic encephalopathy (HIE)

Animal model/Cell line	HBO therapy	Method/Duration	Neurogenesis	Infarct size	Neurobehavioral testing	Mechanism	Institute	Reference
HIE rats	Within 3 h 100%, 2 ATA 1 h/d × 7	BrdU 50 mg/Kg 2 days before sac Q8hX5T	SVZ ↑Brdu+/nestin+ D3, D7, D14 after ischemia	/	/	Wnt-3	Central South University, China	Wang, et al [4]

HIE rats	Within 3 h 100%, 2 ATA 1 h/d × 7	brdU 50 mg/Kg 1 days after surgery Q12hX6D	SVZ ↑Brdu+/nestin+ D3, D7, D14 after ischemia	/	/	MBP	Central South University, China	Yang, et al [11]

HIE rats	Within 3 h 2 ATA, 100% oxygen 1 h/d × 7	brdU 50 mg/Kg 1 days before HBO Q12hX6D	↑D7 SVZ brdu+/DCX+ ↑D14 cortex brdu+/DCX+ D28 cortex ↑BrdU/GFAP/tubulin	/	/	/	Central South University, China	Wang, et al [5]

neural stem cell from HIE rats	Within 2 h 2 ATA, 100% oxygen × 1 h	/	differentiate into ↑neurons ↑oligodendrocytes ↓astrocytes	/	/	β-catenin,	Central South University, China	Zhang, et al [6]

**Table 2:** Vascular dementia

Animal model/Cell line	HBO therapy	Method/Duration	Neurogenesis	Infarct size	Neurobehavioral testing	Mechanism	Institute	Reference
vascular dementia (ligation of Bilateral CCA)	100%,2 ATA, 2 h/d × 10d	/	piriform cortex (Pir) ↑DCX+, Nestin+	/	Shuttle box testing	/	Third Military Medical University	Zhang et al [12]

**Table 3:** Permanent MCAo

Animal model/Cell line	HBO therapy	Method/Duration	Neurogenesis	Infarct size	Neurobehavioral testing	Mechanism	Institute	Reference
Permanent MCAo	100%,2.5 ATA, 1.5 h From 15 min, 1.5 h, 3 h after MCAo	/	D7 ↑GFAP+	↓	garcia	/	University of Leipzig, Germany	Gunther, et al [13]

**Table 4:** Human mesencephalic neural progenitor cell

Animal model/Cell line	HBO therapy	Method/Duration	Neurogenesis	Infarct size	Neurobehavioral testing	Mechanism	Institute	Reference
hmNPCs	100%,1.5 ATA 1 h/d × 5d	Ki67	↑Mature neurons (Tuji, NSE) -GFAP	/	/	HIF-a Stabilizers	University of Leipzig, Germany	Milosevic, et al [7]

Regarding HBOT, most preclinical studies were performed using a rat model of HIE. Wang et al. initiated 7 days of HBOT (2.0 ATA, 100% oxygen, 1 hour daily) starting 3 hours after experimental HIE in rats. Results showed a significantly increased amount of BrdU+/nestin+ cells in the SVZ with a peak at 7 days after HIE [4]. 21 days later, more BrdU+/β-tubulin+ cells were observed in the cortex of treated rats, suggesting that HBOT promotes the proliferation, differentiation and migration of newly generated cells [5].

Our preliminary data shows that HBOT decreases the infarct size with a significantly increased number of BrdU(+) cells in the peri-infarct area 2 week after experimental HIE. We treated operated animals with 1.5 ATA HBO, 100% oxygen once a day for 3 consecutive days. BrdU, dissolved in saline, was injected intraperitoneally (50 mg/kg) 24 hours after HIE, once a day for a total of 7 days.

Furthermore *in vitro *studies suggest that HBOT promotes neural stem cells differentiation into neurons or oligodendrocytes, while inhibiting those stem cells from differentiating into astrocytes [6,7]. HBOT also enhances the proliferation of other supporting cells, including glial cell line-derived neurotrophic nerve growth factor(GDNF) [8] and vascular endothelial growth factor (VEGF) positive cells [8] as well as epithelial cells [9] and human microvascular endothelial cells (HMEC-1) (Table 5) [10].

**Table 5:** effect of HBOT on other type of cells

Animal model/Cell line	HBO therapy	Method/Duration	Proliferation	Infarct size	Neurobehavioral testing	Mechanism	Institute	Reference
experimental spinal cord injury	Right after injury 100%,2.5 ATA 2 h	D7	↑GDNF(+)cell ↑VEGF(+)cell	↓TTC	↑BBB locomotor scale	↓myeloperoxidase (MPO), tumor necrosis factor-a (TNFa) and interleukin-1b (IL-1b)	Taipei Medical University	Tai, et al [8]

Normal rats	60%, 1 ATA 3 days	Brdu Single injection	germinative zone ↑Epithelial Cells	/	/	Oxidative stress?	Washington University	Shui et al [9]

HMEC-1	100%, 2.4 ATA 1 h	/	↑HMEC-1 proliferation	/	/	antioxidant, cytoprotective genes upregulation	University of Connecticut, USA	Godman et al [10]

## Hypothetical mechanisms

Numerous *in vivo *and *in vitro *studies confirm that HBOT induces neurogenesis [5-7,10-13] however, underlying mechanisms remain unknown. Activation of several signaling pathways and transcription factors have been suggested to play an important role in HBOT induced neurogenesis, including Wnt, hypoxia-inducible factors (HIFs) and cAMP response element-binding (CREB).

**Figure 1:**
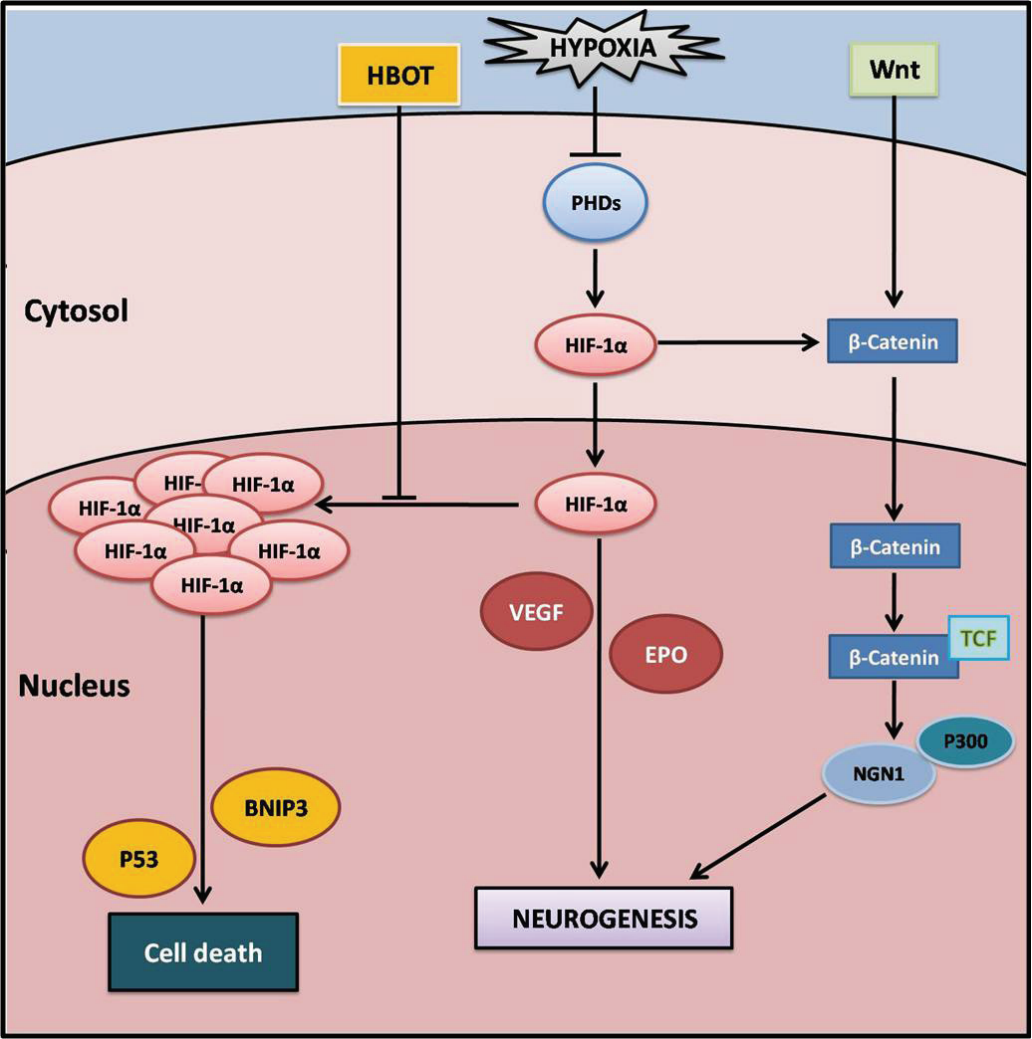
**Potential mechanisms of HBOT and HIF-1α**. In hypoxia, HIF-1α activates EPO and VEGF to promote neurogenesis. The accumulation of HIF-1α further induces the expression of p53 protein and BNIP3, leading to cell death. HBOT stabilizes HIF-1α, preventing it from overexpression and further, activates the Wnt pathway. Abbreviation: PHDs, prolyl hydroxylase; HIF-1α, hypoxia-inducible factor 1α; EPO, e rythropoietin; VEGF, vascular endothelial growth factor; NGN1, Neurogenin1; TCF, T-cell factor.

HIF-1 is a heterodimeric transcriptional complex composed of an inducible HIF-1α subunit and a constitutive HIF-1β subunit. HIF-1α is the principal mediator of cellular hypoxia adaptations [14]. Therefore activated by hypoxia, HIF-1α causes the transcription of its regulated downstream genes, including erythropoietin (EPO) and VEGF which are known to promote neurogenesis [15]. However accumulation of HIF-1α induces expression of p53 [16] and BNIP3 [17], leading to neuronal cell death. Thus neuroprotection may occur shortly after cerebral ischemia at balanced levels of HIF-1α. In the presence of oxygen and iron, HIF-1α is rapidly degraded via the prolyl hydroxylase pathway. Javorina et al. discovered that HBOT exposure stabilizes HIF-1α levels in hmNPCs and furthermore induces neurogenesis *in vitro *[7]. We suggest that HBOT prevents the accumulation of HIF-1α and therefore exerts its neuroprotective effect (Figure 1).

Wnt signaling has been suggested to play an important role in the regulation of cell proliferation and differentiation during the stage of CNS development. Wnt-3 is the starting protein of this pathway. Wang et al. confirmed increased level of Wnt-3 in HBOT rats 3 days after HIE induction, which was positively correlated with the proliferation of stem cells [4]. The authors suggest that cell proliferation via Wnt pathway is regulated through β-catenin. Furthermore, *in vitro *studies demonstrated that β-catenin siRNA decreases the amount of newly generated neurons by repressing the Neurogenin1 (NGN1) gene, which can be reversed by HBOT [6]. It has been recently reported that HIF-1α modulates Wnt/β-catenin signaling in hypoxic embryonic stem cells (ESC) by enhancing β-catenin activation, and expression of the downstream effectors lymphocyte enhancer factor-1 (LEF-1) and T-cell factor-1 (TCF-1) [18].

It has been implicated that Hif-1α deletion reduces Wnt/β-catenin signaling in the SGZ, causing impaired Wnt-dependent processes, including neural stem cell proliferation, differentiation and neuronal maturation [18]. We conclude that activation of the Wnt pathway may occur via HBOT induced control of HIF-1α (Figure 1).

**Figure 2:**
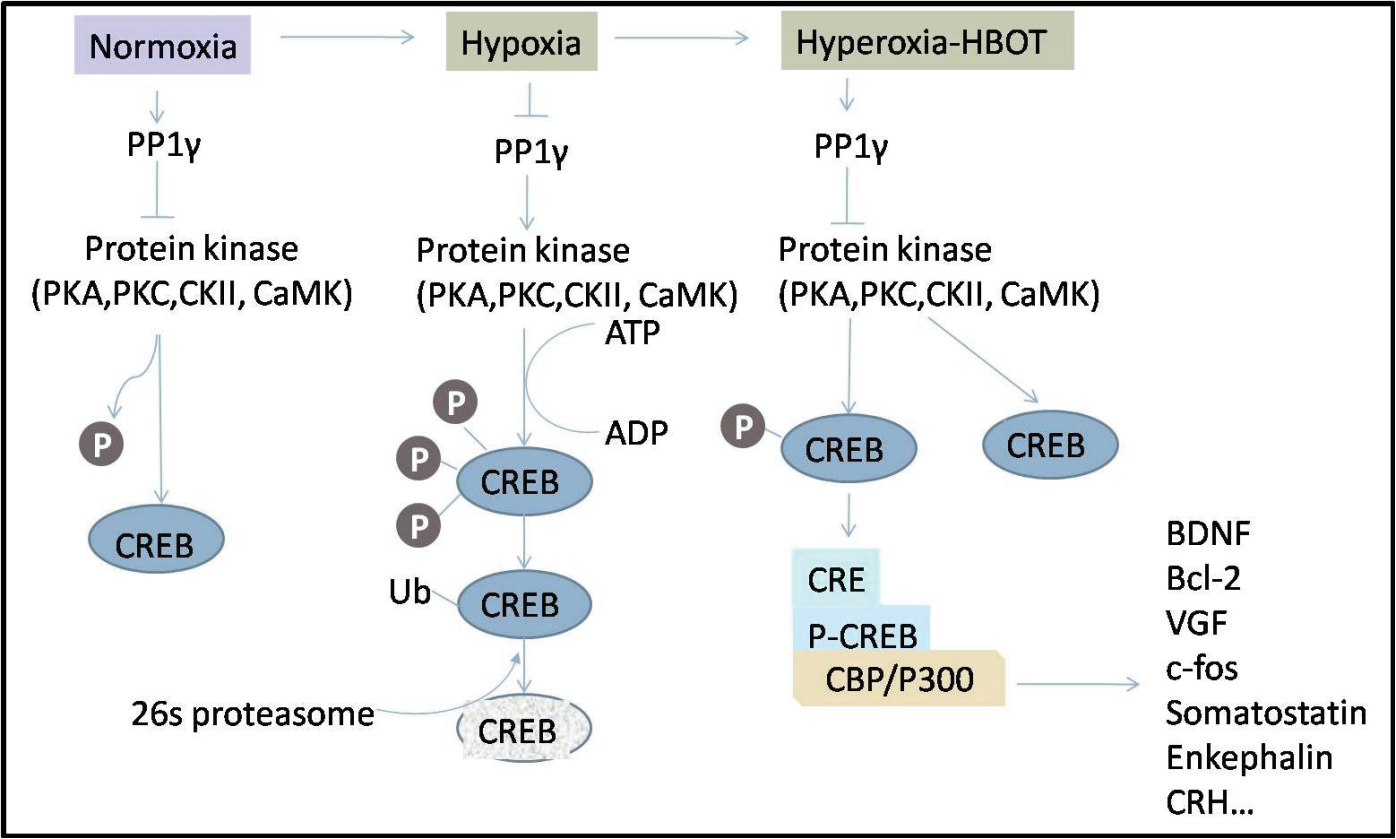
**Potential mechanisms of HBOT associated CREB activation and degradation**: CREB activates its downstream genes when phosphorylated, while PP1 catalyzes the dephosphorylation of CREB. PP1γ is the core subunit of PP1. In hypoxia, PP1γ is repressed, leading to over-phosphorylation of CREB, followed by CREB ubiquitination and degradation by 26s proteasome. HBOT may reverse this process by reactivating PP1γ and blocking CREB degradation. Thus, phosphorylated CREB activates the downstream genes (BDNF, Bcl-2, VGF, et al.) to promote neurogenesis. Abbreviation: PP1γ, protein phosphatase-1; CREB, cAMP response element-binding; Ub, ubiquitin; CRE, cAMP response elements; CBP, CREB binding protein.

CREB plays a well-documented role in neuronal plasticity and formation of long-term memory, mainly through up-regulation of its downstream genes including brain derived neurophic factor (BDNF), Bcl-2, c-fos and VGF. Activation of CREB increases neurogenesis in the DG after focal cerebral ischemia in rats, and protects against hypoxic brain injury [19]. Application of 100% oxygen increased CREB expression in striatum and hippocampus in a neonatal piglet model of intermittent apnea [20]. HBO preconditioning furthermore increased the ratio of Bcl-2 and Bax expression in a MCAo/reperfusion model [21]. CREB activates its downstream genes when phosphorylated, while protein phosphatase-1 (PP1) catalyzes the dephosphorylation of CREB. PP1γ modulates the localization and/or activity of PP1. Suppressed in hypoxic conditions, PP1 leads to over-phosphorylation of CREB, followed by CREB ubiquitination and degradation by 26s proteasome [22]. Although the exact role of CREB in HBOT induced neurogenesis is still not clear, we suggested that HBOT could reverse this process by reactivating PP1γ and by blocking the degradation of CREB (Figure 2).

## Clinical applications

### Stroke

Neurons are highly energy demanding, a characteristic which makes them vulnerable to decreased cerebral blood supply during stroke. Experimental transient ischemia induces neurogenesis in the DG, with a peak between 7-10 days [23]. In confirmation to these results Shin et al. found the highest number of Brdu+ cells in the SVZ, subependymal zone, cortex and striatum 1 week after MCAo [24]. Thus endogenous neurogenesis after ischemic stroke occurs early and is short-lived.

HBOT appears to be a potent method of oxygen delivery [25]. It increases the oxygen partial pressure within the blood and enhances restoration of oxygen supply after ischemic stroke [26]. Previous studies provide evidence that HBOT promotes neurogenesis [4-6,11], reduces infarct size [27,28] as well as hemorrhagic transformation [29] and improves neurological function, in animal models of ischemic stroke [28].

In contrast to these preclinical results no benefit of HBOT was found in stroke patients [30] and HBOT did not improve the clinical outcome in patients 6 months after acute stroke [31]. However, Singhal concluded that HBOT might extend the time window and increase the efficiency of FDA approved r-tPA thrombolysis after acute ischemic stroke [25].

Most clinical trials presented small sample sizes, undifferentiated stroke types, diverse time windows and varying application of HBOT. To bridge the gap between basic science and clinical studies, large scale, well designed, randomized controlled clinical trials are needed to examine the effects on HBOT in terms of acute sensorimotor and chronic cognitive function in patients.

### Traumatic brain injury (TBI)

It has been established that injury-induced neurogenesis contributes greatly to post-injury recovery. After TBI, hippocampal progenitors are activated and result in increased amount of newly generated neurons within the DG [32]. Although there is no literature available on the HBOT induced neurogenesis in preclinical TBI models, HBOT has been applied to TBI patients. The use of HBOT for TBI remains controversial. McDonagh et al., concluded that there was insufficient evidence to establish the effectiveness of HBOT in the treatment of TBI [33]. Rockswold et al., on the other hand, found that HBOT might be potentially beneficial for severe TBI patients [34]. The safety of HBOT was also evaluated and it was pointed out that, if given at proper paradigms, like 1.5 ATA for 60 minutes, HBOT will not cause oxygen toxicity [34]. In a review of available treatments for acquired brain injury (ABI), including TBI, HBOT was suggested with strong level of evidence among non-pharmacological interventions of ABI. Furthermore, HBOT positively improved mortality with level 1 evidence [35]. Laboratory experiments on HBOT induced neurogenesis are needed to investigate the efficiency of HBOT on TBI.

### Autism

Autism is a neuro-developmental disorder associated with hypoperfusion to several areas of the brain, defects of neurogenesis and neuronal migration [36]. The first multicenter, randomized, double-blind, controlled trial in 2009 found that 40-hour HBOT of 24% oxygen at 1.3 ATM produced significant improvement in children's overall functioning, receptive language, social interaction, eye contact, and sensory/cognitive awareness compared to those received slightly pressurized room air [37]. Another study in 2010 on 16 autism patients, adopting a similar treatment paradigm, showed no effect on a wide array of behavioral evaluations [38]. Basic research is needed regarding neuroprotective effects of HBOT and neurogenesis.

## Special concerns

### HBOT and malignancy

It has been previously suggested that neurogenesis occurs within an angiogenic niche, where neurogenesis is closely associated with vascular recruitment and subsequent remodeling [39]. Therefore HBOT may also stimulate angiogenesis by enhancing the proliferation of fibroblasts, epithelial cells and blood vessels [40].

Concerns have been raised whether HBOT promotes the proliferation of cancer cells. To date, there is little evidence that HBOT causes malignant growth or metastasis. A history of malignancy should therefore not be considered as a contraindication for HBOT [40].

### HBOT and oxidative stress

HBOT enhances the production of reactive oxygen species (ROS) and causes oxidative stress in body tissues [10]. Excessive accumulation of oxidative stress may contribute to neurodegenerative processes and cell death in the brain, as seen in diseases like Alzheimer's disease (AD) and Parkinson's disease (PD) [41]. Since HBOT-induced oxidative stress is directly proportional to both exposure pressure and duration, the benefits of HBOT, may outweigh the side effects due to the phenomenon of hormesis. Hormesis is a process that results in a functional improvement of cellular stress resistance, survival, and longevity in response to sub-lethal levels of stress. We suggest that this process might be beneficial in the treatment of oxidative stress associated neurodegenerative diseases like AD and PD.

## Conclusions and future directions

Abounding evidence has shown that HBOT promotes neurogenesis. Future investigations need to be extended to models of neurological diseases, including subarachnoid hemorrhage (SAH), cerebral hemorrhage, AD, PD, surgical brain injury (SBI) and autism for cell proliferation, survival and differentiation. Furthermore, studies need to be conducted to explore whether HBOT induced neurogenesis leads to a functional improvement followed by large scale, strictly controlled clinical trials to establish HBOT as a prevention and/or treatment modality for neurological diseases.

## Conflicts of Interest/Disclosures

The authors declare that they have no competing interests.
